# Trends and Patterns in Bicycle Injuries: The Significance of Protective Equipment

**DOI:** 10.7759/cureus.71437

**Published:** 2024-10-14

**Authors:** Graysen Pitcher, Andrew McCague, Austin Henken-Siefken

**Affiliations:** 1 Surgery, Western University of Health Sciences, Pomona, USA; 2 Trauma and Acute Care Surgery, Desert Regional Medical Center, Palm Springs, USA; 3 Surgery, Desert Regional Medical Center, Palm Springs, USA

**Keywords:** bicycle accident, bicycle crash, bicycle helmet, bicycle injuries, bicycle protective

## Abstract

Introduction

This research presents a five-year comprehensive analysis of trends and efficacy related to helmet and protective equipment use in bicycle-related incidents. Our areas of comparison included injury severity, hospital stay, the Glasgow Coma Scale (GCS) scores, and demographic data. In light of the current increase in bicycle use popularity, this study aimed to provide clear information regarding the significance of protective equipment to combat the surge in bicycle-related incidents in recent years.

Methods

A retrospective analysis was performed using the National Trauma Data Standard (NTDS) as a data source. Our inclusion criteria considered the mechanism of injury involving bicycle incidents, narrowing our pool of patients to 135,605. These cases were screened based on the following parameters: use of helmet/protective equipment, demographic criteria, and several other injury markers. Statistical analyses, including two sample t-tests, were performed using SAS.

Results

A total of 5.7 million patients were screened for inclusion criteria, yielding 135,605 cases involving bicycle-related injuries between 2018 and 2022. Of the patients who met the inclusion criteria, patients wearing helmets had an overall high GCS score (p<0.001) and decreased hospital stay (p<0.001) compared to those without helmet use. Similar findings were drawn related to protective equipment use and GCS (p<0.001). The Injury Severity Score (ISS) on average was higher for those wearing helmets (p<0.001). Overall, an increase in helmet use was noted for the years in question (R^2^=0.467). Additional observations made include large variations in helmet use in different age groups. Patients aged <17 years wore helmets in just 6,514 (22%) cases while those aged +65 years wore helmets in 10,925 (51%) cases.

Conclusions

This study observed a statistically significant difference between GCS values in helmet/non-helmet-wearing groups. Similar findings were found regarding other protective equipment use as well. A statistically significant difference was also found regarding increased ISS with helmet use. This challenges our current assumptions about improved injury severity with the use of protective equipment. In line with other studies, we determined that helmet use tends to decrease the level of injuries on the head. Overall, helmet use has increased in the past five years, but several demographic groups were found to wear protective equipment less often. We recommend future research that focuses on understanding the trends in protective equipment use among bicycle-riding individuals under the age of 25 years, Native Americans, and African Americans. Continued research regarding injury reduction interventions is of utmost importance, especially given the widespread increase in the popularity of bicycle use.

## Introduction

Cycling has attracted remarkable interest in recent years worldwide as a means of transportation as well as a recreational hobby. While there is substantial evidence indicating the benefits of cycling for health and longevity [1], the recent surge of new cyclists on roads and trails alike warrants an interest in the level of safety associated with these activities. Although there has been a steady improvement in protective equipment usage and integration of bicycle lanes into public transportation infrastructure, bicycle-related incidents have constituted a common presentation to the emergency department in the past five years. According to records from 2021, there were an estimated 41,165 bicycle-related injuries (compared to 38,886 in 2020) and a 1.9% increase in fatal pedal cyclist injuries (966) [2]. This noteworthy increase warrants further investigation into the mechanisms and patterns associated with these types of incidents. 

A series of public health measures have been implemented in response to this increase in bicycle-related injuries. Many cities nationwide have implemented the use of bicycle lanes throughout main travel routes. These clearly marked lanes provide a protected space for cyclists to travel without the added concern of sharing road space with other automotive vehicles [3]. Although there are some concerns regarding the efficacy of bicycle lane use, most of the evidence in recent years indicates a reduction in injury severity for bicycle lane users compared to those without [4]. Other safety measures that have been implemented include increased promotion of helmet use, distribution of helmets to low-income families, and improved signage for bicycle-heavy traffic areas. 

A large public health focus has been placed on safe bicycle use with respect to commuter networks. However, in some regions of the United States, mountain biking and bicycle jumping are growing in popularity. Between 2006 and 2017, the number of mountain biking athletes rose from 6.75 million to 8.6 million [5]. These domains of the sport carry their own inherent risks. One systematic review has discussed the environmental factors contributing to mountain biking incidents, and the top contributing factors include slippery terrain, excessive speed, and situational judgment errors [5]. In response to environmental factors, many cities have constructed and maintained a series of public-access bicycle parks with professionally maintained trails and obstacles. These parks aim to provide a more safe and controlled environment for those participating in these activities. While not perfect, these changes are just a few in the list of examples that aim to provide a safe place for cyclists of all skill levels, aimed at a reasonable reduction of incident occurrence.

Large-scale studies published in the past decade have continued to reinforce the accepted standard that protective equipment use by bicycle users decreases the risk of injury and severity of injury in case of accidents. One large-scale meta-analysis reported a 48% reduction in head injuries in bicycle-related incidents [6]. In response to the rising popularity of biking, the bicycle industry has developed and promoted the use of updated safety equipment including helmets, knee pads, elbow pads, and neck braces suited for some riding domains. For example, recent advancements in helmet technology integrate multiple-direction impact protection systems (MIPS) as a means to greatly reduce the risk of brain injury with oblique angle impacts to the head.

Studies suggest that these systems greatly reduce rotational strain associated with several types of impacts [7]. Riders participating in mountain biking or similar activities are encouraged to wear protective equipment like knee pads and elbow pads to minimize impact injuries associated with falls. With the widespread promotion of updated protective equipment along with greater public awareness of safe bicycle practices, we conducted this study to analyze and determine the relative changes in bicycle incident injury mechanisms, the overall use of protective equipment, and the nationwide changes in injury trends in the context of the growing popularity of biking. Additionally, we also sought to determine the changes in the Glasgow Coma Scale (GCS) and Injury Severity Score (ISS) associated with protective equipment use among bicycle users.

## Materials and methods

A retrospective analysis was performed involving patients presenting to trauma centers nationwide in the period 2018-2022. The data were gathered from the National Trauma Data Standard (NTDS) as a de-identified data set. Patients are considered for inclusion in this data set if they have sustained a traumatic injury within 14 days of presentation to the hospital. Patients meeting these criteria are then screened by the International Classification of Diseases, Tenth Revision, Clinical Modification (ICD-10-CM). Patients with a diagnostic code in the range of S00-S99, T07, T14, T79.A1-T.79.A9 are considered for inclusion. Patients are then further screened for one injury code outside of the ranges S00, S10, S20, S30, S40, S50, S60, S70, S80, and S90.

Following this screening process, the lethality of injury, hospital transfer to an acute care facility, and hospital admission are all determined [8]. With over 5.7 million total NTDS patients in our study period, we screened according to their mechanism of injury, with inclusion criteria of 46 injury codes directly pertaining to pedal bicycle incidents. The keywords for our inclusion criteria were "pedal bicycle, pedal bicycle driver, pedal cyclist, and unspecified pedal cyclist." All other injury codes were excluded from our filtered data (Table 1). Following separation by injury code, we identified the following for all patients: age, sex, race, use of protective equipment, use of helmet, ISS, GCS, ED discharge disposition, and total length of hospital stay. Patient data meeting inclusion criteria were filtered and compiled into a separate data set for further analysis.

**Table 1:** All ICD-10 codes included in our criteria for bicycle-related incidents from NTDS data service Total number of codes included in our criteria: 46 ICD-10: the International Classification of Diseases, Tenth Revision; NTDS: the National Trauma Data Standard

ICD code inclusion	
V10.0XXA	Pedal cycle driver injured in collision with pedestrian or animal in nontraffic accident, initial encounter
V10.2XXA	Unspecified pedal cyclist injured in collision with pedestrian or animal in nontraffic accident, initial encounter
V10.4XXA	Pedal cycle driver injured in collision with pedestrian or animal in traffic accident, initial encounter
V10.9XXA	Unspecified pedal cyclist injured in collision with pedestrian or animal in traffic accident, initial encounter
V11.0XXA	Pedal cycle driver injured in collision with other pedal cycle in nontraffic accident, initial encounter
V11.2XXA	Unspecified pedal cyclist injured in collision with other pedal cycle in nontraffic accident, initial encounter
V11.4XXA	Pedal cycle driver injured in collision with other pedal cycle in traffic accident, initial encounter
V11.9XXA	Unspecified pedal cyclist injured in collision with other pedal cycle in traffic accident, initial encounter
V12.0XXA	Pedal cycle driver injured in collision with two- or three-wheeled motor vehicle in nontraffic accident, initial encounter
V12.2XXA	Unspecified pedal cyclist injured in collision with two- or three-wheeled motor vehicle in nontraffic accident, initial encounter
V12.4XXA	Pedal cycle driver injured in collision with two- or three-wheeled motor vehicle in traffic accident, initial encounter
V12.9XXA	Unspecified pedal cyclist injured in collision with two- or three-wheeled motor vehicle in traffic accident, initial encounter
V13.0XXA	Pedal cycle driver injured in collision with car, pick-up truck or van in nontraffic accident, initial encounter
V13.2XXA	Unspecified pedal cyclist injured in collision with car, pick-up truck or van in nontraffic accident, initial encounter
V13.4XXA	Pedal cycle driver injured in collision with car, pick-up truck or van in traffic accident, initial encounter
V13.9XXA	Unspecified pedal cyclist injured in collision with car, pick-up truck or van in traffic accident, initial encounter
V14.0XXA	Pedal cycle driver injured in collision with heavy transport vehicle or bus in nontraffic accident, initial encounter
V14.2XXA	Unspecified pedal cyclist injured in collision with heavy transport vehicle or bus in nontraffic accident, initial encounter
V14.4XXA	Pedal cycle driver injured in collision with heavy transport vehicle or bus in traffic accident, initial encounter
V14.9XXA	Unspecified pedal cyclist injured in collision with heavy transport vehicle or bus in traffic accident, initial encounter
V15.0XXA	Pedal cycle driver injured in collision with railway train or railway vehicle in nontraffic accident, initial encounter
V15.2XXA	Unspecified pedal cyclist injured in collision with railway train or railway vehicle in nontraffic accident, initial encounter
V15.4XXA	Pedal cycle driver injured in collision with railway train or railway vehicle in traffic accident, initial encounter
V16.0XXA	Pedal cycle driver injured in collision with other nonmotor vehicle in nontraffic accident, initial encounter
V16.2XXA	Unspecified pedal cyclist injured in collision with other nonmotor vehicle in nontraffic accident, initial encounter
V16.4XXA	Pedal cycle driver injured in collision with other nonmotor vehicle in traffic accident, initial encounter
V16.9XXA	Unspecified pedal cyclist injured in collision with other nonmotor vehicle in traffic accident, initial encounter
V17.0XXA	Pedal cycle driver injured in collision with fixed or stationary object in nontraffic accident, initial encounter
V17.2XXA	Unspecified pedal cyclist injured in collision with fixed or stationary object in nontraffic accident, initial encounter
V17.4XXA	Pedal cycle driver injured in collision with fixed or stationary object in traffic accident, initial encounter
V17.9XXA	Unspecified pedal cyclist injured in collision with fixed or stationary object in traffic accident, initial encounter
V18.0XXA	Pedal cycle driver injured in noncollision transport accident in nontraffic accident, initial encounter
V18.2XXA	Unspecified pedal cyclist injured in noncollision transport accident in nontraffic accident, initial encounter
V18.4XXA	Pedal cycle driver injured in noncollision transport accident in traffic accident, initial encounter
V18.9XXA	Unspecified pedal cyclist injured in noncollision transport accident in traffic accident, initial encounter
V19.00XA	Pedal cycle driver injured in collision with unspecified motor vehicles in nontraffic accident, initial encounter
V19.09XA	Pedal cycle driver injured in collision with other motor vehicles in nontraffic accident, initial encounter
V19.20XA	Unspecified pedal cyclist injured in collision with unspecified motor vehicles in nontraffic accident, initial encounter
V19.29XA	Unspecified pedal cyclist injured in collision with other motor vehicles in nontraffic accident, initial encounter
V19.3XXA	Pedal cyclist (driver) (passenger) injured in unspecified nontraffic accident, initial encounter
V19.40XA	Pedal cycle driver injured in collision with unspecified motor vehicles in traffic accident, initial encounter
V19.49XA	Pedal cycle driver injured in collision with other motor vehicles in traffic accident, initial encounter
V19.60XA	Unspecified pedal cyclist injured in collision with unspecified motor vehicles in traffic accident, initial encounter
V19.69XA	Unspecified pedal cyclist injured in collision with other motor vehicles in traffic accident, initial encounter
V19.81XA	Pedal cyclist (driver) (passenger) injured in transport accident with military vehicle, initial encounter
V19.88XA	Pedal cyclist (driver) (passenger) injured in other specified transport accidents, initial encounter
V19.9XXA	Pedal cyclist (driver) (passenger) injured in unspecified traffic accident, initial encounter

After data extraction, a two-sample t-test using a hypothesized mean difference of 0 and an alpha value of 0.05 was used to determine if the differences in ISS were statistically significant between groups of patients wearing helmets/no helmets and protective equipment/no protective equipment. We then performed separate two-sample t-tests using a hypothesized mean difference of 0 and an alpha value of 0.05 to draw a comparison between the GCS of patients with/without helmet use and with/without protective equipment use. Likewise, we performed a two-sample t-test using an identical hypothesized mean difference and alpha value to draw a correlation between the total days of hospital stay and protective equipment/helmet use. Patients with no reported information on helmet use, ISS, or GCS were excluded from their respective statistical analyses. All statistical analyses including relevant t-tests were performed using SAS software. 

In addition to t-tests performed, demographic information was gathered from our complete data set. The NTDS uses a numerical value of 1 to indicate positive helmet use and a 0 to designate no helmet use. An identical pattern is followed for protective equipment use and respective racial classification. Our compiled data set was analyzed for helmet use in comparison to racial categories using filtered counts of helmet and non-helmet-wearing individuals in their respective racial categories. Filtered counts of helmet and protective equipment use were also gathered according to age classification, and sex. 

## Results

**Figure 1:**
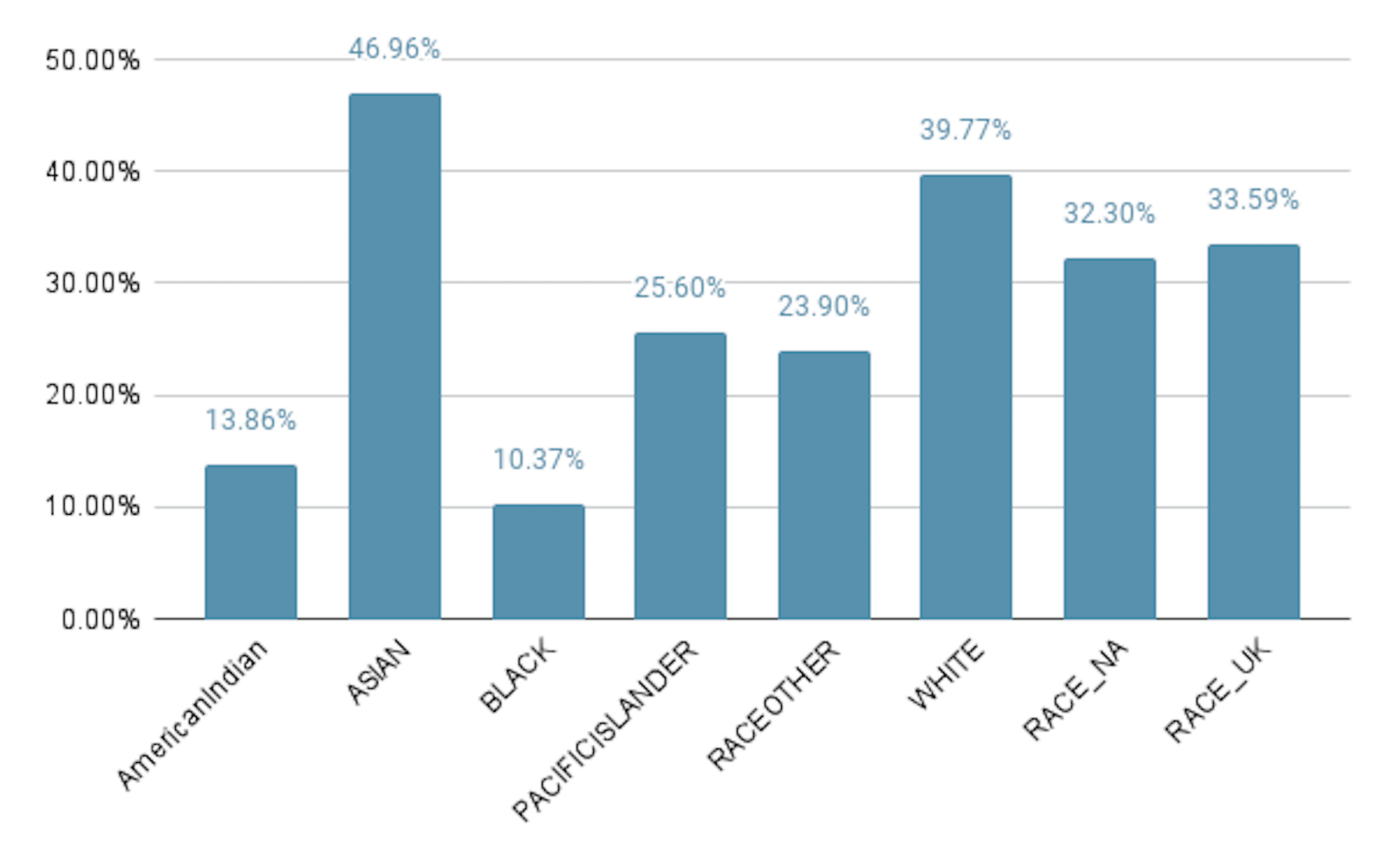
Comparison of individuals in different racial categories based on helmet usage upon presentation due to bicycle-related incidents

**Figure 2:**
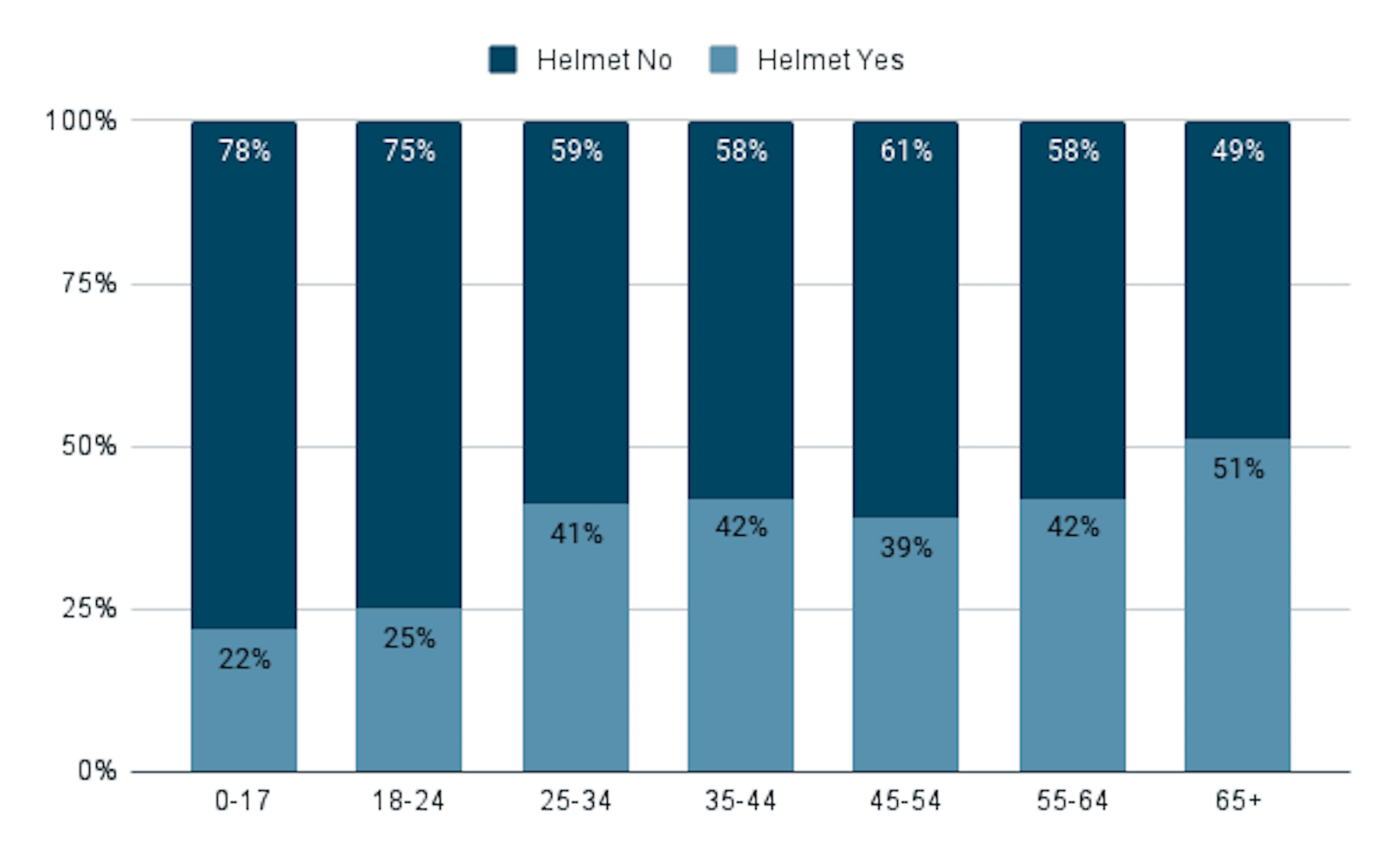
Comparison between helmet users and non-helmet users by age group

The NTDS was exhaustively screened for data relating to bicycle incidents. We screened 5.7 million patients, excluding all patients who did not have a primary injury code related to bicycle incidents; 135,605 patients met our eligibility criteria. Demographic information from our data-driven analysis revealed that most individuals involved in bicycle incidents were white, and the average age of patients was 41.6 years. There was also variation noted in helmet usage among different racial categories. Among Asian individuals involved in bicycle incidents, 1,903 (46.9%) wore helmets, while among black individuals, 1,368 (10%) wore helmets, and among Native American individuals, 135 (13.9%) wore helmets (Figure 1). Additional demographic observations revealed that helmet use varied significantly between age groups; 6,514 (22.3%) of individuals below the age of 18 were noted to be wearing helmets at the time of their incident, compared to 6,912 (40.5%) of those aged 25-34 years, and 10,925 (51%) of those over the age of 65 years (Figure 2).

A comparative analysis between the GCS score and the use of helmets was performed for the years in question. This revealed a statistically significant difference between helmet users and non-helmet users (p<0.001). Other comparisons were made between the ISS and the helmet use and revealed statistically significant differences between the two groups (p<0.001). The observed ISS between helmet users and non-helmet users revealed helmet users had an average ISS of 9.96 and those without helmets had 9.63 (Table 2).

**Table 2:** A representative comparison between several metrics of helmet and non-helmet users as per the NTDS criteria *Statistically significant GCS: Glasgow Coma Scale; ISS: Injury Severity Score; NTDS: National Trauma Data Standard

	No helmet	Helmet	P-value
Number of patients	87,911	48,236	-
Median age, years	38.9	47.5	<0.001*
Sex (M)	71,067	38,436	-
Sex (F)	16,591	9,714	-
Mean ISS	8	9	<0.001*
Mean GCS score	14	15	<0.001*
Mean length of hospital stay, days	4.87	4.01	<0.001*
Total mortality	171	33	-

We also explored several characteristics of populations wearing protective equipment and involvement in bicycle incidents. The mean ISS of individuals wearing protective equipment was noted to be 10.6 compared to 9.7 for those without any (p=0.0006). The average GCS for those with protective equipment was noted at 14.68 and that for those without protective equipment was noted at 14.3 (p<0.001). Additional statistical significance was noted between the hospital stay length between protective equipment and non-protective equipment users. The average hospital stay for those admitted following bicycle incidents with protective equipment use was 4.03 days compared to 4.57 days for non-protective equipment users.

**Figure 3:**
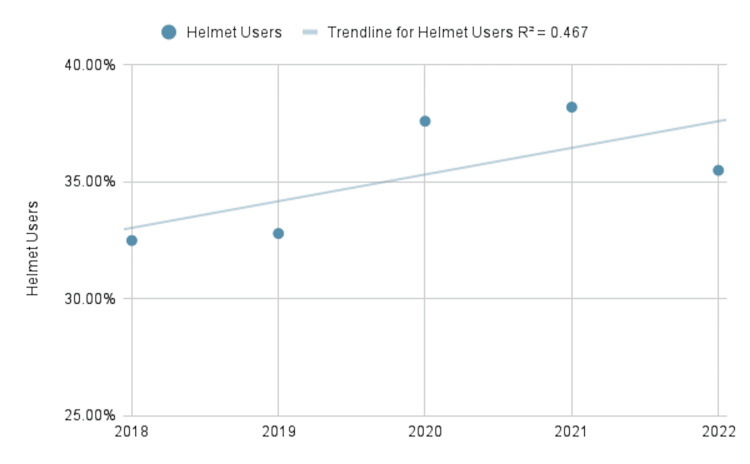
Visual representation of the total proportion of helmet users found in the NTDS for the years 2018-2022 R^2^=0.467 NTDS: National Trauma Data Standard

The analysis of our data also determined that the overall number of injuries associated with bicycle incidents has increased since 2018. In 2018 there were 23,987 total bicycle-related injuries meeting inclusion criteria for the NTDS vs. 27,051 in 2022. It should be noted that the total number of bicycle-related incidents meeting criteria peaked in 2020 at 31,025. Overall, the percentage of patients wearing helmets has increased between 2018 and 2022, with 7,789 (32%) of patients wearing helmets in 2018 compared to 9,606 (36%) in 2022. The overall trend-line for helmet use in the years 2018-2022 reveals a positive growth. The percentage of patients wearing protective equipment in the past five years has remained minimal, with just 772 (<1%) patients presenting to the ED due to bicycle-related injuries wearing protective equipment between the years 2018-2022 (Figure 3).

Our analysis also revealed that a large majority of injuries meeting our inclusion criteria were involved with several particular injury codes. These 130,128 cases accounted for 96% of the total patients. Of these, 69,315 (53%) were involved in traffic-related incidents and 60,813 (47%) were outside of traffic environments (Table 3).

**Table 3:** The 12 most common ICD-10 codes included in our analysis ICD-10: the International Classification of Diseases, Tenth Revision

Common injury codes	N
Pedal cycle driver injured in noncollision transport accident in nontraffic accident, initial encounter	41,993
Pedal cycle driver injured in collision with a car, pick-up truck, or van in a traffic accident, initial encounter	37,081
Pedal cycle driver injured in a noncollision transport accident in a traffic accident, initial encounter	20,594
Pedal cycle driver injured in collision with fixed or stationary object in nontraffic accident, initial	7,007
Pedal cyclist (driver) (passenger) injured in unspecified traffic accident, initial encounter	4,917
Pedal cycle driver injured in collision with a fixed or stationary object in a traffic accident, initial encounter	4,231
Pedal cycle driver injured in collision with car, pick-up truck, or van in nontraffic accident, initial encounter	4,145
Pedal cyclist (driver) (passenger) injured in unspecified nontraffic accident, initial encounter	3,931
Pedal cycle driver injured in collision with another pedal cycle in nontraffic accident, initial encounter	1,942
Pedal cyclist (driver) (passenger) injured in other specified transport accidents, initial encounter	1,795
Pedal cycle driver injured in collision with another pedal cycle in a traffic accident, initial encounter	1,469
Pedal cycle driver injured in collision with heavy transport vehicle or bus in traffic accident, initial	1,023
	130,128

## Discussion

The modern-day discussion regarding the use of helmets in scientific literature provides clear input. The use of helmets plays a significant role in the reduction of brain injury risk, head injury, and upper facial injury [6]. This current understanding is supported by a wide range of research methods including meta-analysis, case studies, and controlled trials. In 2021, a group of researchers proposed that updated helmet technologies improve the overall outcomes for individuals involved in head impacts while riding bicycles. Their study analyzed the rotational forces associated with oblique head impacts at a speed of 6.3 m/s. Their study supported the notion that updated helmets reduce head and brain injury while undergoing oblique angle impacts by reducing the strain placed on the neck and several regions of the brain [7]. Although the amount of research supporting the use of other protective equipment is less abundant, it is clear that the overall body of research suggests a decrease in injury severity following the use of protective equipment. Some sources claim up to an 82% prevention in elbow injuries while wearing elbow pads, and a 32% reduction in knee injuries with the use of knee pads [9].

Another study independently analyzing several case-control studies involving bicycle incidents and helmet use concluded that helmets can provide between a 63% and 88% reduction of brain injury. Their analysis also suggested that this reduction in injury severity also occurs regardless of motor vehicle involvement [10]. Because motor vehicle incidents account for such a large portion of bicycle-related incidents, a large body of research has been created regarding the topic. The overwhelming response in the literature supports the use of bicycle helmets for the reduction of head injuries in motor vehicle-related collisions. One such study performed in Australia even suggested that the more severe the injury considered in motor vehicle incidents, the greater the injury reduction when bicycle users wore helmets [11].

Interestingly, some argue bicycle helmet use may encourage more dangerous behavior. This hypothesis, which is termed the risk compensation hypothesis, has been highlighted in several different sports domains. However, current research suggests that there is no association between an increase in risky behavior and wearing protective equipment [12]. Additionally, some utilize the risk compensation hypothesis to make a stand against helmet legislation or strong advocates of helmet use [13].

Our performative analysis regarding the data in the NTDS highlighted a few interesting supporting details regarding the consensus in scientific literature. It was noted that there is a strong statistical correlation between the use of helmets and an improved GCS score. This data stands to support the wide range of research advocating for the use of helmets [14,15,16]. Interestingly, the GCS scores of individuals wearing protective equipment were also noted to be greater, on average, than those who were not wearing protective equipment. 

One noteworthy observation from our analysis compared the injury severity score between helmet users and non-helmet users. Although there were considerable statistically significant observations within our data (p<0.001), it was noted that the average injury severity score for helmet users was marginally greater than for those with no helmet use. Although seemingly counterintuitive in the light of current standards and understanding in the literature, this observation highlighted several important moderating factors. First, the NTDS does not provide any information for individuals who are involved in bicycle accidents without Emergency Department involvement. Considering the general information in the scientific literature, we think it plausible that helmet usage does indeed decrease injury severity as a population whole, which is not completely represented in the context of this study. Second, there may be errors in reporting the usage of helmets to the emergency department. Situationally dependent, it is logical to think there are some missed individuals wearing helmets not presented in the NTDS. 

It is noteworthy, however, to recognize that the number of people presenting to the emergency department following bicycle-related incidents wearing helmets and protective equipment has increased in the past 5 years. In 2018, 32.5% of patients in our inclusion criteria wore helmets compared to 35.6% in 2022. This suggests a positive overall response to the promotion of safe bicycle practices nationwide. While the population as a whole is responding positively to increased safety measures, several demographic groups would benefit from added initiatives to promote helmet and protective equipment use. For the years in question, just 22.3% of individuals between 0-17 years old wore helmets, and 25% of those between 18-24. This stands in large contrast to the 41% of individuals between the ages of 25-34 that were wearing helmets.

Additionally, several racial groups wore helmets less frequently. Native American patients in our data set were reported to wear helmets 13.9% of the time, and Black individuals in the data were reported to wear helmets in 10.4% of bicycle-related trauma incidents. With consideration of both racial and age demographics, these findings highlight high-potential target groups for the promotion of helmet and protective equipment use. While differences in helmet use between demographic groups are likely multifactorial, possible influencing factors include cost, style, comfort, peer pressure, or a perceived lack of need. Although this study design did not provide the potential to assess these modulating factors, further research into such would provide valuable insight for targets to improve helmet use in target populations. 

In addition, it was noted that a large portion of accidents occur outside of traffic environments. Of the 12 most common incident mechanisms (n=130,128), 47% of those occurred outside of traffic environments. This accounted for 60,813 of the most common contributing incident mechanisms. Interestingly, public health initiatives tend to focus on traffic-related accident prevention. Although these incidents still account for a majority of crashes, these findings support the notion that public health initiatives should be broadened to aim for intervention toward the prevention of both traffic and non-traffic-related incidents. Some studies suggest efficacy in targeted intervention by counseling patients regarding safe off-road riding practices. Healthcare providers with a knowledge of patient hobbies are in a unique position to provide insight to guide injury prevention practices [17].

This study aimed to take a wide-scale analysis of the bicycle-related injuries associated with trauma cases presenting to the ER. It should be noted, however, that this data does not include all individuals who were involved in bicycle accidents. Patients who do not present to the emergency department within 14 days of initial injury are excluded from the NTDS and thus were not included in our study. Additionally, measurement errors in reporting data to this data service can occur. Certain patient presentations may omit particular information like if a helmet or protective equipment was being used at the time of the injury. Additionally, our inclusion criteria did not include any individuals on electric-powered bicycles. This was, in part, due to the unique differences in speed that electric bicycle users encounter. This change in speed could account for altered injury severity in comparison with non-electric bicycle users. However, considering the rapidly rising popularity of electric bicycles, this will be a distinct group worth analyzing in future studies. In regards to safety equipment, the NTDS does not provide information regarding the models of helmets used or their condition. This poses a concern as our study design may not clearly determine if patients were presenting with outdated equipment that provides a decreased level of injury prevention. 

Our retrospective analysis of data from 2018-2022 made it evident that protective equipment and helmet usage does seemingly lead to less severe incidents. This was supported by statistically significant comparisons between GCS and total hospital stay for both protective equipment and non-protective equipment-wearing individuals. 

## Conclusions

Our exploration of the use of protective equipment and helmet use in recent years has generally yielded positive results. NTDS data from the years 2018-2022 revealed particularly significant trends between the use of all protective equipment and the GCS scores of patients meeting inclusion criteria. Our analysis also uniquely revealed that ISS in patients meeting our criteria was on average, higher in protective equipment-wearing individuals. This analysis also identified nationwide gaps in the use of protective equipment suggesting that targeted approaches to improve bicycle safety within particular racial and age groups may greatly enhance protective equipment use and thus injury prevention. While acknowledging gaps in data, this discussion reaffirms the notion that improved helmet and protective equipment use can result in a reduction of injury for many.
